# Dynamics, Diversity, and Virulence of *Aeromonas spp*. in Homestead Pond Water in Coastal Bangladesh

**DOI:** 10.3389/fpubh.2021.692166

**Published:** 2021-07-09

**Authors:** Abdus Sadique, Sucharit Basu Neogi, Tanvir Bashar, Marzia Sultana, Fatema-Tuz Johura, Saiful Islam, Nur A. Hasan, Anwar Huq, Rita R. Colwell, Munirul Alam

**Affiliations:** ^1^icddr, b, Formerly International Centre for Diarrhoeal Disease Research, Bangladesh, Dhaka, Bangladesh; ^2^EzBiome Inc., Gaithersburg, MD, United States; ^3^Center for Bioinformatics and Computational Biology, University of Maryland, College Park, MD, United States; ^4^Maryland Pathogen Research Institute, University of Maryland, College Park, MD, United States; ^5^Johns Hopkins Bloomberg School of Public Health, Baltimore, MD, United States

**Keywords:** *Aeromonas*, coastal pond, seasonality, diversity, toxigenic genes, virulence

## Abstract

Aeromonads are aquatic bacteria associated with frequent outbreaks of diarrhea in coastal Bangladesh, but their potential risks from environmental sources have remained largely unexplored. This study, over 2 years, examined homestead pond waters in the region for monthly dynamics and diversity of *Aeromonas* spp. The bacterial counts showed bi-modal annual growth peak, pre- and post-monsoon, strongly correlating (*p* < 0.0005) with temperature. Of 200 isolates characterized, *Aeromonas veronii* bv. sobria (27%) was predominant among co-existent *Aeromonas schubertii* (20%), *Aeromonas hydrophila* (17%), *Aeromonas caviae* (13%), and three more. PCR screening of virulence-related genes identified 15 genotypes (I to XV), however, enterotoxigenicity in animal model was observed for five genotypes, ca. 18% (nine of 50) strains, prevalent in *A. veronii* bv. sobria, *A. hydrophila*, and *A. caviae*. Pathogenic strains were distinguishable by possessing at least three of the major virulence genes: *ascV, hlyA, ela, ast*, and *alt*, together with accessory virulence factors. PFGE of *Xba*I-digested genomic DNA revealed high genetic diversity and distant lineage of potentially toxigenic clones. Therefore, along with increased global warming, *Aeromonas* spp. having multi-factorial virulence potential in coastal ponds that serve as drinking water sources pose a potential health risk, and underscores the need for routine monitoring.

## Introduction

The coastal region along the Bay of Bengal, Bangladesh is considered to be one of the most vulnerable areas of the world where climate change mediated impacts are imminent (1). Saline intrusion into the shallow aquifers of this region is a serious problem for millions of rural residents who are vulnerable to waterborne diseases since the ponds serve as their source of water for household purposes, including drinking (2). Recurrent diarrhea and enteric diseases cause serious health problems for the coastal residents, especially children under the age of five. All of the etiologic agents in this disaster-prone region have not been determined. Aeromonads are among the aquatic bacteria associated with water and food-borne diseases in tropical regions (3). These aquatic bacteria are also implicated as causative agents of a variety of disease conditions in aquatic animals, including economically important prawns and fishes. In the central inland areas of Bangladesh, *Aeromonas* spp. have been reported to be associated with gastroenteritis, with a yearly isolation rate between 9 and 16% of stool samples collected from patients with diarrhea (4). However, there is a lack of information regarding the seasonal abundance, diversity of pathogenic potential, and the molecular traits of *Aeromonas* spp. in the coastal region of the Bengal delta.

Members of the genus *Aeromonas* (family Aeromonadaceae) are Gram-negative, rod-shaped bacteria, ubiquitously occurring in various aquatic environments, including freshwater ponds and lakes, sewage waters and brackish waters (5, 6). *Aeromonas* spp. are capable of withstanding a broad range of environmental conditions, e.g., different salinity levels, wide temperature range (4–45°C) and nutrient-limited conditions (7, 8). Changes in the physico-chemical parameters of the coastal waters may modulate abundance of *Aeromonas* populations and the seasonality of their disease incidence. Gastroenteritis caused by *Aeromonas* spp. in humans include mild and self-limiting watery diarrhea, chronic diarrhea lasting more than a year and more invasive *Shigella*-like severe dysentery. Health disasters and life-threatening conditions have been reported, notable in young (under 5 years of age) children and travelers (9, 10). Patients infected with aeromonads generally have stools characterized as watery or containing mucus without visible blood and the majority manifest symptoms that include vomiting and abdominal pain, very similar to the classical symptoms of cholera (11). In addition, *Aeromonas* spp. are associated with extra-intestinal infections in humans, such as life-threatening septicemia, necrotizing fasciitis, hemolytic-uremic syndrome, meningitis, myonecrosis, and serious lung infections (12). *Aeromonas* spp. was ranked as the predominant pathogen (20% the isolates) causing skin or soft tissue infections, among Tsunami survivors (13).

Of the 36 known species of *Aeromonas*, the most common species isolated from clinical samples include *A. hydrophila, A. caviae* and *A. veronii* biovar (bv.) sobria (*A. sobria*), while 15 other species, including *A. salmonicida, A. schubertii, A. trota*, and *A. media* are also associated with human disease (10, 14). A variety of virulence factors of *Aeromonas* spp. have been characterized, for example, hemolysin (*hlyA*), cytotonic heat-labile (*alt*) and heat-stable (*ast*) enterotoxins, Type II-secreted cytotoxic enterotoxin (*act*), Type III secretion system (*ascV*), cytotoxic heat-labile enterotoxin referred to as aerolysin (*aerA*), flagella (*fla*), lipase (*lip*), elastase (*ela*), and protease (*pro*) (15–19). Among the pathogenic factors of *Aeromonas* spp., secretion of cytotonic and cytotoxic enterotoxins encoded by *ast, hlyA*, and *act* genes, and elastase (*ela*) are considered to be the major virulence determinants linked to diarrhea in experimental animals, e.g., mice and rabbits (15, 20). Type III secretion system (TTSS) has been also reported to induce diarrhea (21). Both *hlyA* and *aerA* genes encode for hemolysis of red blood cells and damage to tissue culture cell lines (20, 22). Accessory pathogenic factors include extracellular secretion of protease and elastase, associated with host tissue damage and facilitating nutrient availability and resistance to host immune response (23–25). Some strains of *Aeromonas* produce lipase during invasion of host organs, facilitating lysis of host erythrocyte plasma membranes (24). Cytotonic enterotoxins *ast* and *alt* do not cause degeneration of crypts and villi of the small intestine as observed for the cytotoxic enterotoxins *act* and *aerA* (15). Interestingly, the roles of genes encoding for various pathogenic factors in *Aeromonas* have not been determined in detail and pathogenesis, with respect to *Aeromonas*, is considered to be complex and multi-factorial (10). In this context, aquatic *Aeromonas* populations in the coastal environment of Bangladesh merits investigation. The objective of this study was to determine the spatio-temporal dynamics and diversity of potentially virulent *Aeromonas* spp. in homestead pond waters commonly used by resident people for household purposes in the coastal region of Bangladesh.

## Methods

### Study Area and Field Sampling

**Figure 1:**
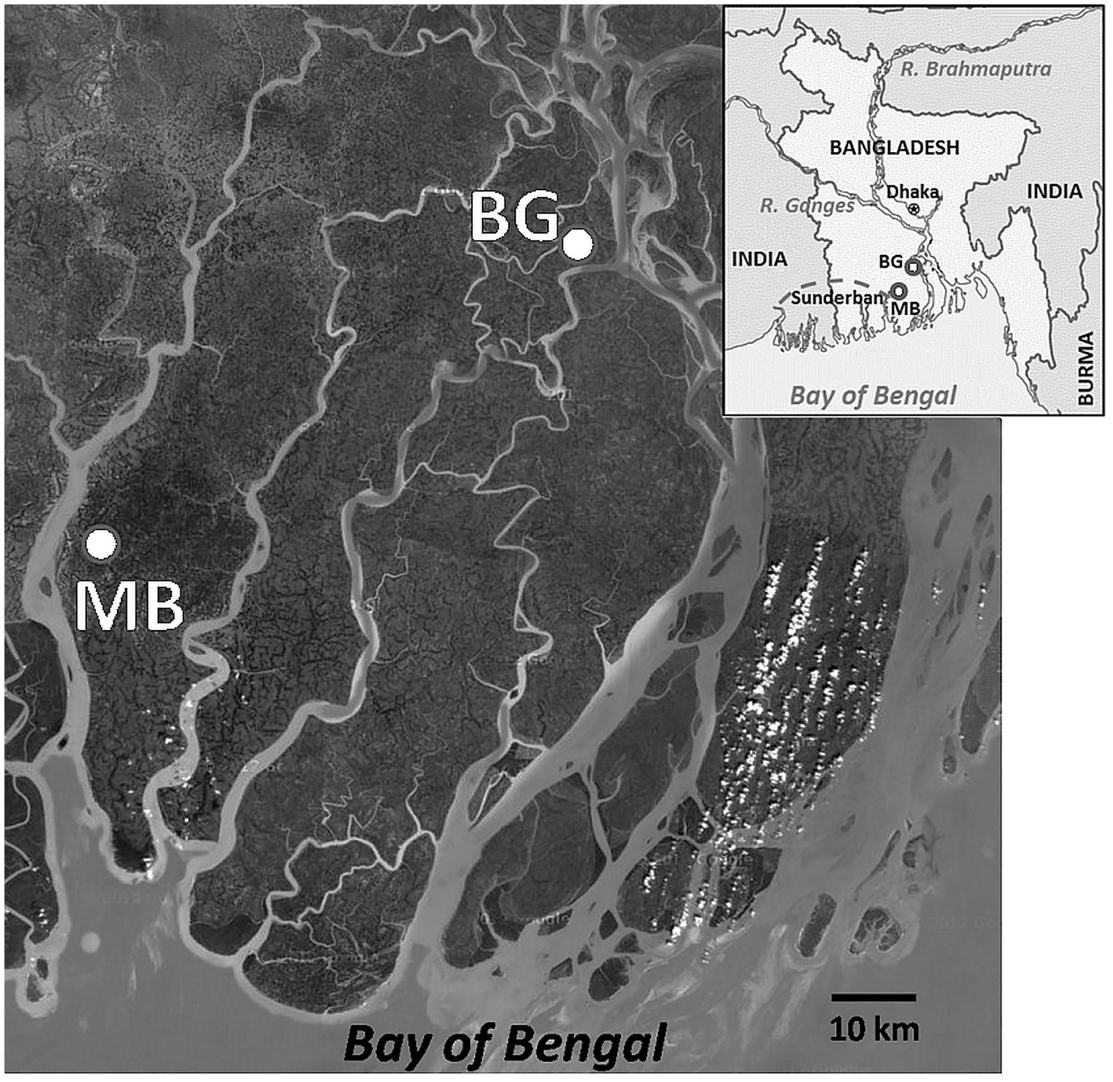
A geographical view of the study areas in the coastal villages of the Bengal delta, Bangladesh. Location of study sites, Mathbaria (MB) and Bakergonj (BG), are shown by closed circles. Inset showing a map of Bangladesh, including areas where water samples were collected from the coastal zones of the Ganges-Brahmaputra river basin. Locations of Dhaka, the capital of Bangladesh, and Sundarban, the largest mangrove wetland in the world, are also shown. This has been taken from Google Map.

Water samples were collected each month during June, 2005 to September, 2007 from two homestead ponds (~0.2 ha each and 2–3 m depth), one in coastal Mathbaria and the other in Bakerganj districts of Bangladesh (Figure 1). Both ponds are located near a tidally influenced tributary of the Ganges-Brahmaputra river system. Located along the western boundary of Mathbaria is the “Sundarban,” the largest tropical mangrove forest in the world. The Bakergonj study site is located ~50 km upstream of Mathbaria. During each sampling five sub-samples of sub-surface water (ca. 0.5 m depth) were collected in a sterilized bucket from each of the four corners and at the middle point of the ponds and pooled. The samples, transported in 1.0 L sterile plastic bottles in an insulated plastic box, were collected between noon to 2.0 p.m. and processed within 18 h of collection. Temperature, pH, salinity, conductivity and dissolved oxygen (DO) of sub-surface water were measured at each sampling, employing a Sens-Ion III multi parameter device (HACH Co. Loveland, Cleveland, USA), according to manufacturer's instruction.

### Isolation and Identification of *Aeromonas* spp.

A 100 ml portion of each of the water samples were filtered through a 0.22 μm polycarbonate membrane filter (Millipore Corp., Bedford, MA, USA), and the concentrated particulates, including bacteria on filter was re-suspended in 2.5 mL phosphate-buffered saline (pH 7.5) for microbiological analysis. The concentrated bacteria in water samples (100 μL fractions) were cultured on Ryan's *Aeromonas* medium and TCBS agar medium (Difco Laboratories, Detroit, MI, USA) and incubated at 37°C for 24–48 h. In addition, a 1 mL portion of the concentrated water sample was enriched in 9 mL alkaline peptone water (Difco) at 37°C for 6 to 8 h before plating on selective media, as described elsewhere (5).

For each sample, 10 colonies typical of *Aeromonas* spp. were selected and screened for cytochrome oxidase and gelatinase production, susceptibility to O/129 (2,4-diamino-6,7-diisopropylpteridine, 150 μg), and growth at various salt concentrations (0, 6.5, and 8% NaCl). The phenotypically confirmed *Aeromonas* colonies in 100 μL of concentrated water samples was determined by counting colony forming unit (CFU) on selective media. The total count in the original sample were estimated by back calculation with the concentration factor (40X). A total of 200 isolates, including 1–4 representative(s) from each of the *Aeromonas*-positive samples, were identified to species level, employing the Aero key II scheme and other biochemical tests, as described elsewhere (26). All isolates were preserved as glycerol stock, according to standard methods, and stored at −80°C.

### Hemolytic Activity

Representative *Aeromonas* strains were examined for alpha (α) and beta (β) hemolytic activity on blood agar plates, supplemented with 5% de-fibrinated sheep blood, following a previously established method (27).

### Antimicrobial Susceptibility

Antimicrobial susceptibility testing for the isolated strains of *Aeromonas* spp. was performed by disc diffusion method using Muller Hinton agar (Difco) following WHO guidelines (www.who.int/csr/resources/publications/drugresist/en/IIIAMRManual.pdf). Seven different antimicrobial agents (Oxoid, Hampshire, UK) commonly used to treat diarrhea, e.g., tetracycline (30 μg), furazolidone (100 μg), cephalothin (30 μg), erythromycin (15 μg), gentamicin (10 μg), ciprofloxacin (5 μg), and trimethoprim/sulfamethoxozole (1.25/23.75 μg), were used. The diameters of the inhibition zones were interpreted according to the standards for antimicrobial susceptibility testing (28). *E. coli* ATCC 25922 was included for quality control.

### Detection of Virulence Genes

Cells were harvested from overnight cultures of *Aeromonas* strains in Luria-Bertani broth (Difco) and genomic DNA was extracted following standard method as described elsewhere (29). PCR assays were performed using genomic DNA of each strain as template to detect the presence of enterotoxins and virulence-related genes of *Aeromonas* spp., including aerolysin (*aerA*), heat labile and stable enterotoxin (*alt* and *ast*), haemolysin (*hlyA*), Type II-secreted cytotoxic enterotoxin (*act*), TTSS (*ascV*), flagella subunit protein (*fla*), regulatory elastase (*ela*), protease (*pro*) and lipase (*lip*), employing previously described primers and PCR conditions (Table 1). Samples (3 μl) were added to the PCR mixture to achieve a 30-μl final volume containing a 0.21 mM concentration of each deoxynucleoside triphosphate mixture, 50 mM KCl, 1.5 mM MgCl_2_, 10 mM Tris-HCl (pH 8.3), 0.17 μM primer pair and 0.75 U of Taq polymerase (Takara, Kyoto, Japan). Amplification conditions used were 5 min at 94°C for initial denaturation of DNA and 30 cycles, each consisting of 1 min at 94°C, 1 min at 55°C, and 1 min at 72°C, with a final round of extension for 7 min at 72°C in a DNA RoboCycler gradient temperature cycler (Stratagene, La Jolla, Calif.). The PCR products were subjected to 1.5% agarose gel electrophoresis in TAE buffer (40 mM Tris-acetate, 1 mM EDTA), followed by staining in ethidium bromide solution (2 μg ml^−1^) and destaining in distilled water for 5–10 min each. Images were captured by Gel-Doc 2000 (Bio-Rad, Hercules, CA, USA).

**Table 1:** Details of PCR assays targeting virulence genes of *Aeromonas* spp.

**Sl. No**.	**Target gene**	**Primer sequence (5′ to 3′)**	**Amplicon size (bp)**	**References**
1	*ascV*	ATGGACGGCGCCATGAAGTT	710	(21)
		TATTCGCCTTCACCCATCCC		
2	*hlyA*	CCACGCAAATTCATCACG	1,079	(30)
		ATCCTTGTTCACCTCGAC		
3	*ela*	ACACGGTCAAGGAGATCAAC	513	(18)
		CGCTGGTGTTGGCCAGCAGG		
4	*ast*	TCTCCATGCTTCCCTTCCACT	331	(18)
		GTGTAGGGATTGAAGAAGCCG		
5	*alt*	TGACCCAGTCCTGGCACGGC	442	(18)
		GGTGATCGATCACCACCAGC		
6	*act*	AGAAGGTGACCACCAAGAACA	232	(31)
		AACTGACATCGGCCTTGAACTC		
7	*aerA*	CTGGTCTGGATAGACGGGCTCTGCC	416	(32)
		GCCTGAGCGAGAAGGT		
8	*pro*	ATGACTAACCCTTTGCTG	1,038	(30)
		GAACTTGTGCTGCTTGAG		
9	*lip*	ATCTTCTCCGACTGGTTCGG	382	(18)
		CCGTGCCAGGACTGGGTCTT		
10	*fla*	TCCAACCGTYTGACCTC	608	(18)
		GMYTGGTTGCGRATGGT		

The presence of putative virulence genes among various *Aeromonas* strains were also confirmed by colony hybridization. A positively amplified PCR product of each gene was purified after agarose gel electrophoresis using the gel extraction kit (QIAGEN, Hilden, Germany) and labeled with digoxigenin (DIG) using a commercial kit according to manufacturer's instructions (Roche Diagnostics GmbH, Mennheim, Germany). Freshly cultured *Aeromonas* colonies were grown on nitrocellulose membranes (0.22 μm, Millipore Corp.) overlaid on LB Agar (Difco) at 37°C for 4–6 h. The colonies were lysed, using denaturation solution (0.5 M NaOH) for 10 min, followed by exposure three times in neutralization solution (1 M Tris-HCl, pH 7.0) for 1 min each and finally treated with 1 M Tris-HCl (pH 7.0), 1.5 M NaCl for 10 min. The membranes were air-dried and DNA was cross-linked to the membrane by UV irradiation for 5 min. The blots, thus prepared, were subjected to a hybridization with the DIG-labeled PCR products of target genes as probes using the DIG detection kit (Roche Diagnostics GmbH) according to manufacturer's instruction.

### Virulence Potential

The enterotoxigenic potential of selected environmental strains of *Aeromonas* spp. was examined in both the rabbit ileal loop (RIL) and suckling mice assay (SMA). Culture filtrates (CF) of representative *Aeromonas* strains belonging to different species and having different virulence gene profiles were prepared as described previously (27). Culture filtrates of cholera toxin (CT) positive *Vibrio cholerae* 569B and sterile media (Luria Bertani Broth) were used as positive and negative controls, respectively.

The RIL assay was performed using adult New Zealand white rabbits (~2.0 kg) following a previously established method (33). Briefly, 1 ml of CF was injected into each loop of a rabbit ileum, following laparotomy, and the inoculated rabbits were sacrificed after 16–18 h to measure the volume of fluid accumulation per cm of gut in each rabbit. Fluid accumulation of ≥0.5 ml cm^−1^ of gut was considered as a positive response in the RIL.

The SMA was performed using 3 days old Swiss albino suckling mice, according to standard procedures as described previously (34). Briefly, an aliquot (0.1 ml) of the CF containing 0.01% (w/v) Evans Blue was injected directly into the stomach of each suckling mouse and after 4–5 h of incubation the mouse was sacrificed, and its intestine was removed and weighed. A fluid accumulation score, in SMA, was expressed as the ratio of weight of the intestine to the remaining body weight and a ratio of ≥0.08 was considered as positive. Enterotoxigenic potential of each strain was verified in triplicate.

The experimental animals were reared at the animal resources facility of icddr,b in accordance with the Guide for the Care and Use of Laboratory Animals (1996, published by National Academy Press, 2101 Constitution Ave. NW, Washington, DC 20055, USA). The Animal Experimentation Ethics Committee (AEEC) of icddr,b approved the study protocol.

### Genomic Diversity and Clonal Relationship

To determine genomic diversity of the coastal *Aeromonas* populations, representative strains were subjected to pulsed field gel electrophoresis (PFGE). Bacterial cells were harvested from colonies grown overnight at 37°C on Trypticase Soy Agar (Difco), supplemented with 5% defibrinated sheep blood, and suspended in cell suspension buffer (100 mM Tris-HCl, 100 mM EDTA, pH 8.0). The cell suspensions, after adjusting absorbance to ca. 0.64–0.66 at 560 nm, were mixed at 56°C with an equal volume of 1% SeaKem^®^ Gold Agarose (Cambrex Bioscience Rockland, Inc., Rockland, ME, USA) in TE buffer (10 mM Tris and 1 mM EDTA, pH 8.0) and solidified in plug molds (Bio-Rad Laboratories, Richmond, CA, USA). Gel plugs were incubated with cell lysis buffer (50 mM Tris, 50 mM EDTA, and 1% N-lauroyl sarcosine) for 2 h at 55°C with vigorous shaking followed by twice washing in autoclaved distilled water at 50°C for 15 min each and four times washing in TE buffer at 50°C for 1 h each. The gel plug was digested with 20 U of *Xba*I (Invitrogen, Life Technologies, NY, USA) in 200-μl reaction volumes for at least 4 h at 37°C. PFGE was performed using 1% PFGE certified agarose (Bio-Rad) and 0.5X TBE (0.089 M Tris, 0.089 M boric acid, 0.002 M EDTA) running buffer in a CHEF MAPPER (Bio-Rad). Electrophoresis was done at six volts for 18 h (initial switch time 2.2 s; final switch time 63.8 s). *Salmonella braenderup*, a control strain for DNA size markers, was also subjected to PFGE at each step. Following electrophoresis, the gels were stained for 30 min, de-stained twice for 15 min each, and pictures captured using a Gel-Doc 2000 (Bio-Rad).

To determine clonal relationships among the *Aeromonas* strains, PFGE patterns in gel images were subjected to cluster analysis by the unweighted-pair group method using average (UPGMA) and the band-based (Dice coefficient) option was applied during dendogram similarity calculation using BioNumerics (Applied Maths BVBA, Sint-Martens-Latem, Belgium). Pulsotypes were determined considering a 90% cut-off of the similarity index, while estimating their clonal lineage.

### Statistical Analysis

Statistical analyses were carried out using “Xact” (version 7.21d, SciLab, Saint Yrieix, France) and Statistical (ver. 10.0, StatSoft, Oklahoma, USA). Non-parametric Spearman rank correlations were used for the data analysis. Regression fits were applied to explore correlations between variables, including environmental factors and bacterial abundance. A *p* < 0.05 was considered as significant.

## Results

### Physicochemical Parameters

**Figure 2:**
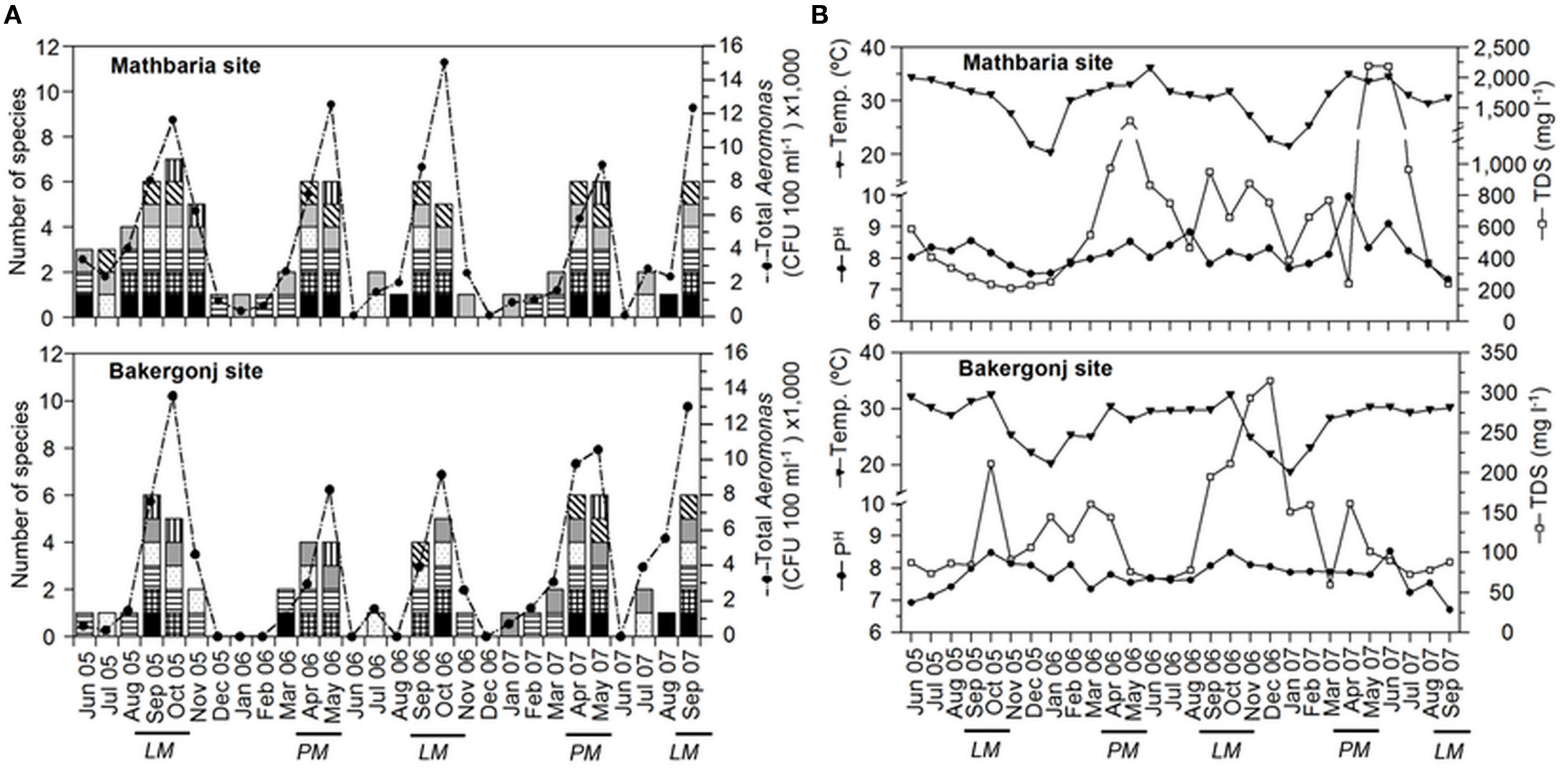
Dynamics of *Aeromonas* populations and physicochemical variables in pond waters in the coastal zone of Bangladesh. **(A)** Changes in total cultivable populations shown as line graph and species diversity, shown as stacked columns, in Mathbaria (top panel) and Bakergonj (bottom panel) sites of coastal Bangladesh. Individual species are demarcated in the stacked columns as: *A. veronii* bv. sobria (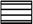), *A. schubertii* (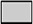), *A. caviae* (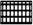), *A. hydrophila* (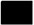), *A. trota* (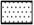), *A. allosaccharophila* (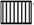) and *A. eucrenophila* (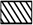). **(B)** Changes in water temperature (Temp.), pH and total dissolved solids (TDS) in Mathbaria (top panel) and Bakergonj (bottom panel) sites. “*PM”* and “*LM”* indicates the “pre-monsoon (spring)” and “late monsoon (autumn)” seasons, respectively.

In both study areas, the water temperature during sample collection ranged from 18 to 25°C during the winter season (December to February) and 25–36°C during the warmer months (March-November). The monsoon season, characterized by frequent precipitation, extended from June to October. The Mathbaria site had comparatively higher salinity (0.2–2.4 practical salinity unit [PSU]) than Bakergonj (0.0–0.3 PSU). Similarly, TDS values were also higher at Mathbaria (190–2,200 mg L^−1^) than Bakergonj (67–195 mg L^−1^), and their seasonal peaks also showed distinct spatial variation (Figure 2). Water pH was relatively higher at Mathbaria (7.3–9.8) than Bakergonj (6.7–8.5). Variation in DO (4.2–9.6 mg L^−1^) was independent of season or site. A clear influence of monsoon rainfall, leading to reduced salinity and TDS was observed at Bakergonj during May-September. However, further downstream from the Mathbaria site, the impact of rainfall was not discernible, probably, because of a significant impact of tidal flushing.

### Seasonal Dynamics of *Aeromonas* spp.

*Aeromonas* spp. were isolated from 82% (46 out of 56) samples, with higher abundance during the summer months (>25°C of water temperature) and low or no culturable counts during winter months (Figure 2). The yearly isolation rate of *Aeromonas* spp. was higher in pond water at Mathbaria, which is located closer to the Bay of Bengal shoreline than Bakergonj pond water (Supplementary Table 1). However, *Aeromonas* counts exhibited a bi-modal pattern annually, reaching >10^4^ CFU 100 ml^−1^, during pre-monsoon spring (April-May) and at the autumn (September-October), at both study sites (Figure 2).

No difference in the *Aeromonas* species diversity was observed between the two study sites. Overall, seven species of *Aeromonas* were identified after screening 200 isolates. Among them, *A. veronii* bv. sobria (27.0%) dominated, followed by *A. schubertii* (19.5%) and *A. hydrophila* (17%), whilst *A. caviae* (13%), *A. trota* (12.0%), and *A. eucrenophila* (7.0%) were isolated intermittently. *A. veronii* bv. sobria and *A. schubertii* were frequently isolated throughout the year, while *A. allosaccharophila* was rarely (4.5%) isolated (Figure 2). Notably, along with the increased occurrence of culturable population, the diversity of *Aeromonas* spp. was significantly higher during pre-monsoon (April-May, late spring/early summer) and post-monsoon (September-October, autumn) time of the year. Seasonal variations in *Aeromonas* species diversity were similar for both of the study sites (Supplementary Table 1).

### Influence of Physico-Chemical Parameters on *Aeromonas* Dynamics

**Figure 3:**
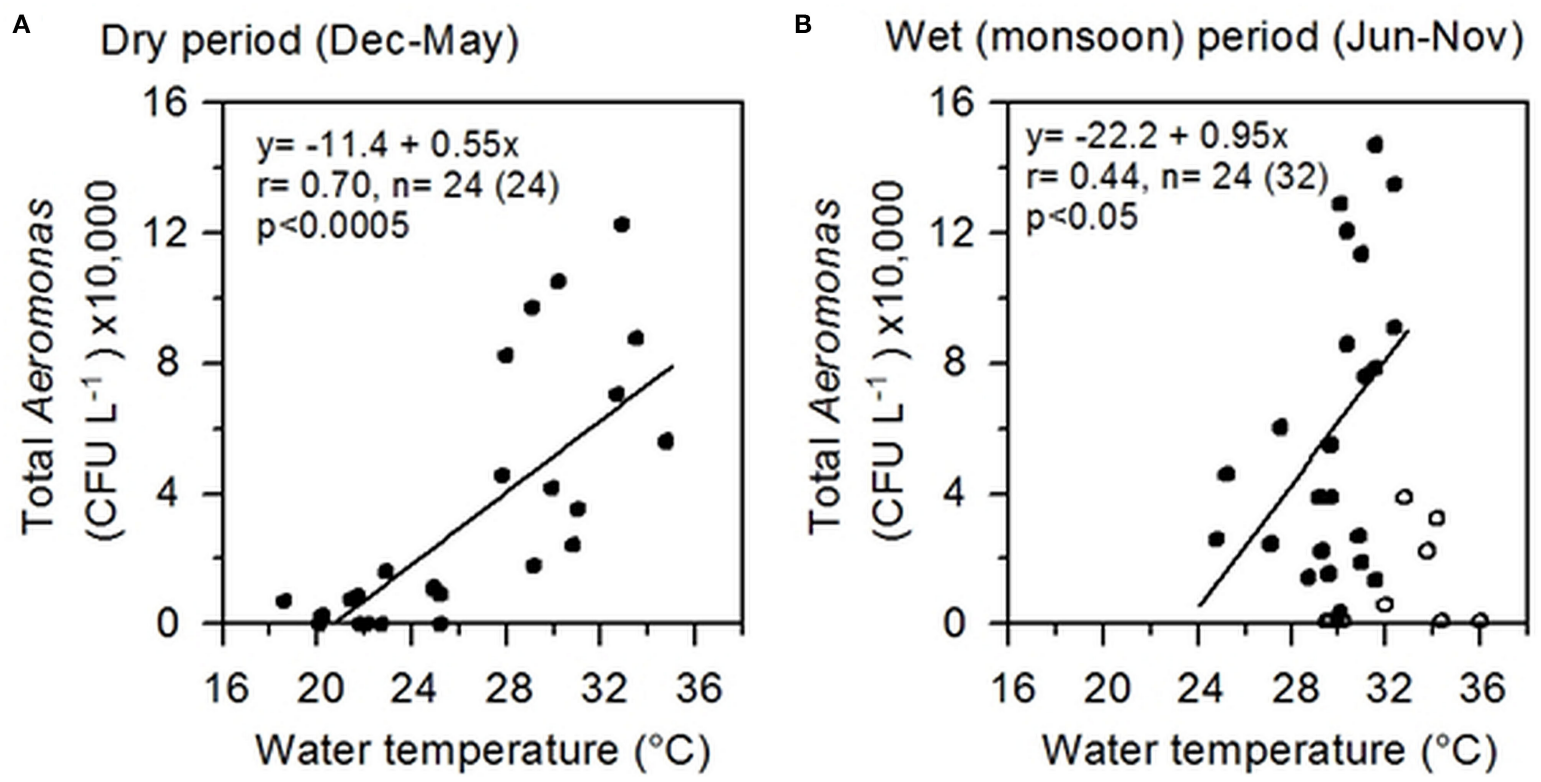
Correlation between *Aeromonas* abundance and water temperature. **(A)** Correlation during the dry period (December to May); **(B)** Correlation during the wet period (June to November), excluding eight outliers. Lines represent linear regressions; relevant information including regression equation, “r” and “p” values are shown inside each box. Correlating data are shown as filled circles (dots) and the outliers as empty circles.

Highly significant (*p* < 0.0005) correlation was observed between water temperature and total culturable *Aeromonas* spp., notably during the dry period (December to May), and at temperatures >25°C, explaining ~70% of variations in bacterial abundance (Figure 3). During the wet monsoon, despite warmer climate, the temperature influence on *Aeromonas* populations was minimized, yet significantly (*p* < 0.05) explaining 44% of the variations in the bacterial culturable populations. Except for water temperature, no other physico-chemical parameters, including pH, temperature, DO, salinity, and TDS, was found to have any significant influence on *Aeromonas* abundance in water bodies.

### Hemolytic Activity

On blood agar, 35 of 50 (74%) *Aeromonas* strains had shown hemolytic activity. The majority (74%) of the strains were β-hemolytic and only seven (14%) were α-hemolytic (Table 2). The α-hemolytic strains included *A. veronii* (*n* = 4), *A. caviae* (*n* = 2), and *A. hydrophila* (*n* = 1). Six strains, including all *A. eucrenophila* (*n* = 3), and one each of *A. schubertii, A. trota*, and *A. allosaccharophila* showed no hemolytic activity.

**Table 2:** Occurrence of virulence related genes and hemolytic activity among the strains of different *Aeromonas* species.

**Species (*n*)**	**Genes related to pathogenicity [*****n*** **(%)]**										Hemolytic activity^*^ [*n* (%)]
	***ascV***	***hlyA***	***ela***	***ast***	***act***	***aerA***	***alt***	***pro***	***lip***	***fla***	
*A. veronii* bv. sobria (14)	3 (21)	4 (29)	5 (21)	5 (36)	7 (50)	8 (57)	11 (79)	10 (71)	12 (86)	13 (93)	4 α (29), 9 β (64)
*A. schubertii* (10)	0 (0)	0 (0)	0 (0)	3 (30)	4 (40)	8 (80)	7 (70)	7 (70)	8 (80)	8 (80)	9 β (90)
*A. hydrophila* (9)	3 (33)	1 (11)	3 (33)	3 (33)	6 (67)	4 (44)	8 (89)	8 (89)	8 (89)	9 (100)	1 α (33), 8 β (89)
*A. caviae* (7)	3 (43)	2 (29)	3 (43)	3 (43)	3 (43)	4 (57)	5 (71)	5 (71)	7 (100)	7 (100)	2 α (29), 4 β (57)
*A. trota* (5)	0 (0)	0 (0)	0 (0)	0 (0)	4 (80)	3 (60)	4 (80)	4 (80)	4 (80)	4 (80)	5 β (100)
*A. eucrenophila* (3)	0 (0)	0 (0)	0 (0)	0 (0)	0 (0)	0 (0)	0 (0)	3 (100)	0 (0)	0 (0)	3 β (100)
*A. allosaccharophila* (2)	0 (0)	0 (0)	0 (0)	0 (0)	1 (50)	1 (50)	1 (50)	2 (100)	1 (50)	2 (100)	1 β (50)
Overall (50)	9 (18)	7 (14)	11 (22)	14 (28)	25 (50)	28 (56)	34 (68)	39 (78)	40 (80)	43 (86)	

*n = 50;*

* *α and βdesignates the alpha and beta hemolytic activities on blood agar medium, respectively*.

### Antimicrobial Resistance Patterns

A total of 50 representative *Aeromonas* strains were screened for their response to seven commonly used antimicrobial agents. Except for *A. veronii* (*n* = 14), all strains of different *Aeromonas* species (*n* = 36) were resistant to Cephalothin. However, all *Aeromonas* strains were found to be susceptible to the remaining six antimicrobial agents tested.

### Occurrence of Toxin Genes

The major virulence determinants, i.e., genes associated with enterotoxigenicity, namely, *ascV, ast, hlyA*, and *ela*, encoding TTSS, cytotonic heat-stable enterotoxin, elastase, and hemolysin, were detected in 18, 28, 14, and 22%, respectively (Table 2). The gene *ast* was infrequently detected in *A. veronii* bv. *sobria, A. hydrophila, A. caviae*, and *A. schubertii*. Gene *hlyA* was detected only in a few of *A. veronii* (*n* = 4), *A. caviae* (*n* = 2), and *A. hydrophila* (*n* = 1) strains, which correlated with the α-hemolytic activity of these strains on Blood Agar. Similarly, the *asc* and *ela* genes were found in a few strains of these three species of *Aeromonas*. The accessory virulence-related genes encoding flagella (*fla*), lipase (*lip*), protease (*pro*), aerolysin (*aerA*), cytotoxic enterotoxin (*act*), and cytotonic heat-labile enterotoxin (*alt*) were detected in 86, 80, 78, 70, 56, and 48%, respectively, of *Aeromonas* strains. However, occurrence of these genes showed variations (between 40 and 100%) in different species (Table 2). *Aeromonas* strains (*n* = 15), which did not show any hemolytic activity, lacked *aerA, act*, and *hlyA*, whereas all strains carrying *aerA* were found to be β-hemolytic on Blood Agar. Overall, occurrence of toxin encoding genes was higher among the strains belonging to *A. veronii* bv. sobria, *A. hydrophila*, and *A. caviae*.

### Distribution and Prevalence of Virulence Genotypes

Approximately 15 “virulence genotypes,” designated as “genotype I” to “genotype XV,” could be differentiated among 50 representative *Aeromonas* strains (Table 3). The first five genotypes (I-V) harbored all or most of the major virulence determinants, i.e., *ascV, ast, hlyA, ela, act, aerA*, and *alt*, in addition to accessory virulence-related genes. Genotype I harbored all virulence-associated genes tested, except *alt*. Genotypes II-V harbored eight virulence genes, whereas genotypes VI- X, XI-XII, XIII-XIV, and XV harbored 5, 4, 3, and 1 of 10 virulence-associated genes, respectively (Table 3). Genotype VII (*act-aerA-pro-lip-fla*), distributed among four species (*A. schubertii, A. veronii* bv. sobria, *A. hydrophila*, and *A. trota*), had the highest prevalence (16%). *A. veronii* revealed greater diversity, in terms of distribution of representative strains into multiple virulence genotypes than other species.

**Table 3:** Distribution, prevalence, and virulence potential of the genotypes detected in *Aeromonas* strains isolated from coastal ponds in Bangladesh.

**Genotype**	**Virulence related genes**										Distribution & occurrence^*^	**Prevalence^†^**	**Virulence potential^§^**
	***ascV***	***hlyA***	***ela***	***ast***	***alt***	***act***	***aerA***	***pro***	***lip***	***fla***	**Species (*n*)**	**(%)**	
I	***+***	***+***	***+***	***+***	***–***	***+***	***+***	***+***	***+***	***+***	**Ac (2)**	**4**	**ET**
II	***+***	***–***	***+***	***+***	***+***	***–***	***+***	***+***	***+***	***+***	**Ah (2)**	**4**	**ET**
III	***+***	***+***	***+***	***+***	***–***	***–***	***+***	***+***	***+***	***+***	**Av (2)**	**4**	**ET**
IV	***+***	***+***	***–***	***–***	***+***	***+***	***+***	***+***	***+***	***+***	**Ah (1)**	**2**	**ET**
V	***–***	***+***	***+***	***–***	***+***	***+***	***+***	***+***	***+***	***+***	**Av (2)**	**4**	**ET**
VI	–	–	–	+	+	–	+	–	+	+	Av (2), Ah (1), Ac (1), As (1)	10	NT
VII	–	–	–	–	–	+	+	+	+	+	As (3), Av (2), Ah (2), At (1)	16	NT
VIII	–	–	–	–	+	–	+	+	+	+	At (2), Ac (2), Av (1), Ah (1)	12	NT
IX	–	–	+	–	–	+	–	+	+	+	Ac (1), Ah (1), Av (1)	6	NT
X	–	–	–	+	–	+	+	+	–	+	As (2), Av (1)	6	NT
XI	+	–	–	–	–	+	–	–	+	+	Ac (1), Av (1)	4	NT
XII	–	–	–	–	+	–	+	+	–	+	At (1), Ah (1), Av (1), Aa (1)	8	NT
XIII	–	–	–	–	–	–	–	+	+	+	As (2), Aa (1)	6	NT
XIV	–	–	–	–	+	+	–	***–***	+	–	As (2), Av (1), At (1)	8	NT
XV	–	–	–	–	***–***	–	–	+	–	–	Ae (3)	6	NT

* *Species names are abbreviated: Ah, A. hydrophila; Ac, A. caviae, Av, A. veronii bv. sobria; As, A. schubertii; Ae, A. eucrenophila; At, A. trota; Aa, A. allosaccharophila*.

† *Prevalence among 50 representative strains of different Aeromonas species*.

§ *ET, capable of producing diarrhea, i.e., enterotoxigenic and NT, not enterotoxigenic, as revealed from animal experiments (rabbit ileal loop and suckling mice assays, Supplementary Table 2). The results of potentially virulent strains of Aeromonas spp. are shown in bold texts*.

### Enterotoxigenic Potential in Animal Model Experiments

Three *Aeromonas* species (18% of the total) belonging to genotypes I to V, i.e., *A. veronii* bv. sobria, *A. hydrophila*, and *A. caviae* accumulated fluid in the experimental rabbit and mouse intestine, and hence concluded to be enterotoxigenic (Table 3). Both SMA and RIL *in vivo* assays yielded similar results (Supplementary Table 2). In RIL experiments, fluid accumulation (FA) ratio ranged between 0.9 and 1.4 ml cm^−1^, while the SMA score ranged between 0.09 and 0.13 for enterotoxigenic strains. However, none of *A. schubertii, A. trota, A. eucrenophila*, or *A. allosaccharophila* induced significant fluid accumulation in both assays. The majority of *Aeromonas* classified as genotypes VI-XV were not enterotoxigenic in mice intestine and rabbit ileal loop assays. Interestingly, *Aeromonas* strains enterotoxigenic in experimental animals were observed to possess multiple virulence genes, including *ascV, ast, hlyA, alt, act*, and *aerA* (Table 3). Among putative accessory virulence factors, selective presence of the *ela* gene was observed among the enterotoxigenic strains, along with co-occurrence of the *lip, fla*, and *pro* genes. Overall, the animal experiments suggest that, without the combined presence of the five major virulence determinants, *ascV-ast-hlyA-ela-alt*, or at least three of them, namely *ascV-hlyA-alt, ascV*-*ast*-*ela*, or *hlyA*-*ela-alt, Aeromonas* strains did not cause fluid accumulation (Supplementary Table 2).

### Genomic Diversity and Clonal Relationship

**Figure 4:**
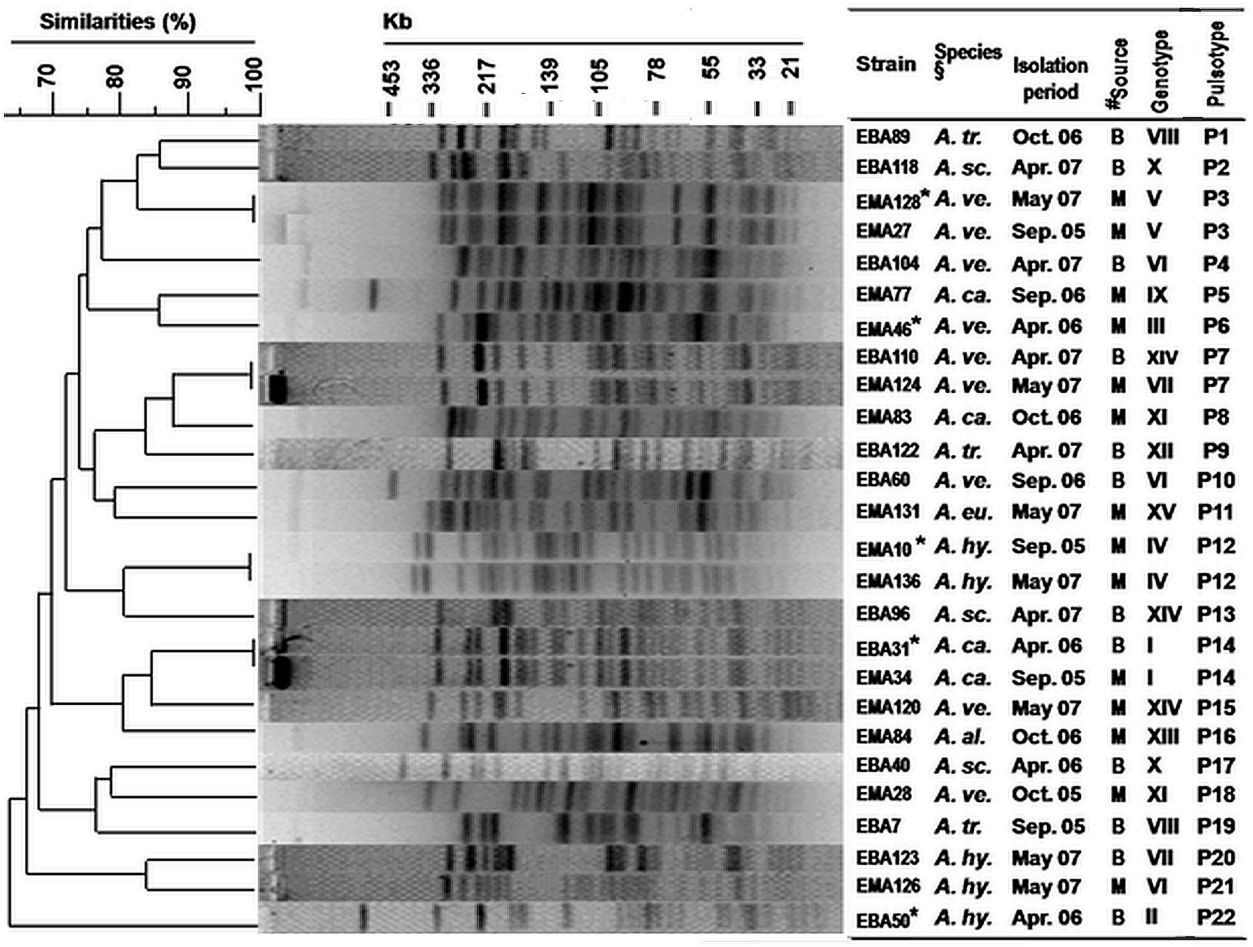
Genetic diversity (by PFGE fingerprinting) among selected *Aeromonas* strains isolated from the pond waters in the coastal zone of Bangladesh. PFGE fingerprints were analyzed by using BioNumerics 4.0 software (Applied Maths Inc., TX, USA). Cluster analysis was done by the unweighted-pair group method using average (UPGMA) linkages and the band-based (Dice coefficient) option was applied. *Strains showing enterotoxigenic activities in rabbit ileal loop and suckling mice assays (Supplementary Table 2). ^§^Species names are abbreviated. ^#^Isolation sources, “B” and “M” represented the coastal sites, “Bakergonj” and “Mathbaria”, respectively.

PFGE analysis of *Xba*I digested chromosomal DNA of representative *Aeromonas* strains yielded 19–28 reproducible DNA fragments, ranging in approximate sizes from <20 to 500 kbp (Figure 4). DNA fingerprints showed high genetic diversity, reflected by the identification of 22 pulsotypes (P1 to P22) of 26 selected strains. Seven distinct pulsotypes were obtained for nine strains of *A. veronii* bv. sobria, and similarly, high genetic divergence was also observed among the other *Aeromonas* species.

All five genotypes were enterotoxic by RIL and SMA. The strains were genetically heteroenous as they clustered into five pulsotypes (P3, P6, P12, P14, and P22). Conversely, identical pulsotype was observed for a few strains belonging to different genotypes (Figure 4).

Overall cluster analysis by dendrogram of the deduced pulsotypes revealed heterogeneity and distant clonal relationship among these coastal *Aeromonas* strains, which included those that were enterotoxigenic (Figure 4). Clonally identical *Aeromonas* strains of pulsotype P3 (enterotoxigenic, genotype V) were detected in both of the years 2005 and 2007, whereas, a dissemination of *A. caviae* strains of pulsotype P14 (enterotoxigenic, genotype I) in both Bakergonj and Mathbaria was remarkable.

## Discussion

This study, to our knowledge, provides the first comprehensive investigation of spatio-temporal dynamics and toxigenic potential for *Aeromonas* spp. in surface water of homestead ponds in the coastal region of Bangladesh. A few earlier studies have explored the ecology of *Aeromonas* spp. in inland waters near Dhaka, in the central part of Bangladesh without coastal influence (5, 6, 35). The study sites: Mathbaria and Bakergonj are located in the coastal region of Bengal delta where diarrheal diseases are prevalent. This is largely because the underground water is saline rich and thus untreated surface waters from river and ponds are used by rural people for household purposes, e.g., washing utensils and vegetables, bathing, gargling, cooking, and drinking (36). The observed occurrences of at least seven *Aeromonas* species including potentially toxigenic strains in homestead ponds indicate the potential threat that they could pose of gastroenteritis among the resident people in coastal villages.

The surveillance carried out during this study period shows that *Aeromonas* populations in these coastal tropical waters have distinct seasonal patterns. The occurrence of two peaks of *Aeromonas*, one during late spring (April-May) before the onset of monsoon and the other during the post-monsoon autumn (September-October), coincides with seasonality of diarrheal incidence in Bangladesh (37). Although *V. cholerae*, enterotoxigenic *E. coli* (ETEC), and rotavirus are commonly identified as major diarrhea causing pathogens in Bangladesh, in ~45% of the cases the causative agent(s) are unidentified (38). In the routine diagnostic procedures for identifying pathogens responsible for diarrhea in Bangladesh, *Aeromonas* spp. is not usually targeted. The observed correlation of seasonal patterns of the incidence of Aeromonas populations in surface water and diarrhea cases in Bangladesh strongly suggest a role of toxigenic aeromonads as one of the causal agents of diarrheal disease in coastal villages of the Bay of Bengal.

Despite the difference in distance from the shoreline, salinity, and other marine related factors, the dynamics of the *Aeromonas* populations are similar, namely recurrence of population peaks in the spring and autumn, in both study areas. Previous investigations reported occurrence of only one summer peak of *Aeromonas* spp., in the inland surface waters of Bangladesh (35). However, a study done on the Italian coast found stochastic variation in aeromonad numbers (10^2^-10^6^ CFU 100 ml^−1^) throughout the year, without a conspicuous seasonality (39). In Bangladesh, plankton blooms often occur in many ponds and lakes during or before the two diarrhea epidemic periods (pre- and post-monsoon). *Aeromonas* spp. can secrete many extracellular enzymes including chitinase, which may aid in bacterial adherence and growth on chitinous particles and plankton such as copepods, shrimps, crabs etc. (24). Degradation of the plankton and other chitin-rich substances supports rapid growth and proliferation of aeromonads and other bacteria present in the water body. In absence of such nutrients during the off-peak seasons, majority of *Aeromonas* populations might persist in the environment in a dormant state in which the bacterium remains viable but non-culturable (VBNC) in conventional laboratory media (40). Upon availability of nutrient under optimum bio-physicochemical conditions during the seasonal peak or when introduced into host intestine, the VBNC bacterial cells can regain culturability and become active again (41).

Temperature dependent variations in the *Aeromonas* populations, correlating with gastroenteritis and extra-intestinal infections caused by these organisms, in aquatic habitats have been observed previously with highest abundance in summer and lowest in winter (3, 8). The present study also observed aquatic *Aeromonas* populations and temperature to be correlated, with strong significance during non-monsoon period and relatively low significance during monsoon. Temperature is a vital influencing factor for many bacterial populations in coastal and oceanic habitats and at higher temperatures (>20°C) bacteria rapidly utilize dissolved organic substrates (42, 43). Among other environmental factors, an increase in salinity up to 10 PSU favors growth of *Aeromonas* spp. (3). In the present study, salinity of coastal pond water was relatively low (0.0–2.4 PSU) and a significant impact of salinity on *Aeromonas* dynamics was not discernible. The greater isolation rate and comparatively higher number of *Aeromonas* spp. in the more saline Mathbaria pond water may be explained by the bacterial better adaptation to brackish water rather than freshwater.

The diversity of cultivable *Aeromonas* species in the study ponds was relatively higher compared to what has been reported previously for inland freshwater and sewage systems of the Bengal delta and coastal areas of Europe where only 3–4 species were reported (5, 6, 39). The dominance of *A. hydrophila, A. veronii* biovar sobria, and *A. caviae* among coastal pond waters is in agreement with the earlier studies. Moreover, the present study shows *A. schubertii* is a commonly occurring species in coastal Bangladesh. Detection of *Aeromonas* species in this study was based on phenotypic characteristics, and this might have underestimated species diversity of cultivable coastal *Aeromonas* populations, including *Aeromonas aquareorum*, which can be misidentified as *A. hydrophila* unless amplification and sequencing of the *gyrB* and *rpoD* genes are performed (44). Moreover, existence of even higher species diversity is expected because bottom sediment and aquatic organisms, including phyto- and zooplankton, which serve as important reservoirs of *Aeromonas* spp. were not included in this study (35, 45). It is evident from the present study that in addition to PFGE analysis, screening of virulence genes may aid in differentiation of strains producing identical pulsotypes (Figure 4). Further PCR analysis targeting recently known accessory virulence factors, ADP-ribosyltransferase toxin (*aexT*) and DNases (*exu*), may provide additional discriminatory results (46). The observed occurrence of identical virulence genotype in several species, or also different virulence genotypes among strains of the same species of *Aeromonas* appear to contradict with the hypothesis that each species possesses a distinct set of virulence genes (46). However, the whole genome sequencing (WGS) remains a future option (47), while other approaches of molecular typing, e.g., Enterobacterial Repetitive Intergenic Consensus (ERIC) PCR, and Repetitive Extragenic Palindromic (REP) PCR, in combination with sequencing of a number of housekeeping (16S rRNA, *gyrB* and *rpoD*) and virulence genes would be required to better understand the inter- and intra-species genetic diversity, clonal lineage, and horizontal gene transfer among the *Aeromonas* populations existing in the aquatic environment.

Results of the present study show that *A. veronii* bv. sobria, *A. hydrophila*, and *A. caviae*, which are the dominant species among the *Aeromonas* populations in coastal homestead ponds in Bangladesh, are potentially enterotoxigenic. This appears in agreement with the frequent occurrence of these three species reported for stool samples from patients suffering from gastroenteritis in Bangladesh (4, 5, 48). Fortunately, the coastal *Aeromonas* strains examined in this study were found to be susceptible to common antimicrobial agents, whereas high incidence of multi-drug resistant *Aeromonas* has been reported in central inland areas of Bangladesh (49).

A correlation between the pathogenic potential of *Aeromonas* and its β-hemolytic activity, induced by the *hlyA* and *act* genes has been noted in previous studies (15, 50). Other *Aeromonas spp*. may produce α-haemolytic activity by cytolysis coded by the *aerA* gene (32). Results of present study support the previous findings. Among the major virulence determinants associated with *Aeromonas* gastroenteritis occurrence of *alt* and *act* genes may synergistically induce more severe diarrhea than the *alt* gene alone (16). In addition, TTSS of *Aeromonas* (*ascV*^+^ strains) plays crucial roles in bacterial pathogenesis by translocation of ADP-ribosylating toxins directly into the host cell to induce apoptosis (21). The present study also shows that the pathogenic potential of *Aeromonas* strains in causing diarrhea greatly depends on a combined presence of the major virulence genes: *ascV, ast*, and/or *hlyA*, and *alt*, along with co-occurrence of accessory virulence-stimulatory genes, *ela, act*, and *aerA*.

The low-altitude coastal zones of the Bengal delta is one of the most vulnerable regions to climatic disasters, including cyclones, extreme temperature and rainfall, and coastal inundations, which have profound influences on the dynamics and diversity of aquatic microorganisms, including pathogenic bacteria (51, 52). In the recent decade, due to increased salinity of groundwater as a consequence of sea level rise, millions of people in coastal Bangladesh has no option but to use contaminated natural surface waters for household purposes including drinking, and therefore, highly vulnerable to waterborne diseases (53). The occurrence of multiple toxigenic genes in approx. one-fifth of the representative *Aeromonas* strains, which could be potentially pathogenic and categorized into at least five virulence genotypes (I-V), in coastal region of Bangladesh, is of great public health implication.

## Conclusion

The present study indicates a potential risk of pathogenic aeromonad populations in surface water of homestead ponds in the coastal region of Bangladesh. The dominant species with enterotoxigenic potential found in this study included *A. veronii* bv. sobria, *A. hydrophila* and *A. caviae*. Molecular characterization of virulence related genes of *Aeromonas* strains isolated from the Bengal delta region showed that they were highly diverse having at least 15 virulence genotypes. Cluster analysis by dendrogram using PFGE images also revealed 22 pulsotypes in 26 *Aeromonas* strains which were highly heterogenous genetically. A combined action of at least three of the major virulence determinants, *ast-ascV-hlyA-ela-alt*, and also indefensible synergistic mechanisms of multiple virulence-associated genes, most likely determine the pathogenic potential of the virulent strains. A community-based epidemiological surveillance in parallel to environmental surveillance is essential to assess potentially virulent environmental genotypes of *Aeromonas* spp., and their correlation with diarrhea incidence in this region. The overall data presented in this study could serve as a baseline for future studies and aid intervention and preventive measures against these epidemiologically significant *Aeromonas* spp. thriving naturally in the aquatic environment.

## Data Availability Statement

The original contributions presented in the study are included in the article/Supplementary Material, further inquiries can be directed to the corresponding author/s.

## Ethics Statement

The animal study was reviewed and approved by icddr,b Animal Experimentation Ethics Committee (AEEC). Written informed consent was obtained from the owners for the participation of their animals in this study.

## Author Contributions

AS and SI: resources and investigation. SN: conceptualization and formal analysis. AS and TB: investigation and writing—original draft preparation. MS: project administration and resources. F-TJ: resources. NH, AH, and RC: writing—review and editing. MA: supervision and writing—review and editing. All authors contributed to the article and approved the submitted version.

## Conflict of Interest

NH and RC are employees of EzBiome Inc. The remaining authors declare that the research was conducted in the absence of any commercial or financial relationships that could be construed as a potential conflict of interest.
