# Supplementary material for: Dynamics, Diversity, and Virulence of Aeromonas spp. in Homestead Pond Water in Coastal Bangladesh
Source: Front Public Health. 2021 Jul 9;9:692166. doi: 10.3389/fpubh.2021.692166 (PMC8298834; doi:10.3389/fpubh.2021.692166)
Supplement: Supplementary file 1 [file Table_1.docx]

**Supplementary Table 1. Isolation of *Aeromonas* spp. from pond water in the coastal zone of Bangladesh***

| Species | 2005 (Jun-Dec) | |  | 2006 (Jan-Dec) | |  | 2007 (Jan-Sep) | | Total |
| --- | --- | --- | --- | --- | --- | --- | --- | --- | --- |
|  | MB | BG |  | MB | BG |  | MB | BG | (%) |
| *A. veronii* bv. sobria | 8 | 7 |  | 10 | 8 |  | 12 | 8 | 54 (27.0) |
| *A. schubertii* | 10 | 4 |  | 11 | 4 |  | 7 | 3 | 39 (19.5) |
| *A. hydrophila* | 8 | 3 |  | 7 | 3 |  | 8 | 5 | 34 (17.0) |
| *A. caviae* | 5 | 3 |  | 6 | 5 |  | 4 | 3 | 26 (13.0) |
| *A. trota* | 4 | 7 |  | 3 | 5 |  | 2 | 3 | 24 (12.0) |
| *A. eucrenophila* | 4 | 0 |  | 6 | 1 |  | 3 | 0 | 14 (7.0) |
| *A. allosaccharophila* | 2 | 2 |  | 1 | 1 |  | 2 | 1 | 9 (4.5) |

*n = 200; MB, Mathbaria site; BG, Bakergonj site

**Supplementary Table 2. Enterotoxigenic potential of representative *Aeromonas* strains isolated from coastal ponds***

| Species | Strain |  | Virulence related genes | | | | | | | | | Genotype | RIL | SMA |
| --- | --- | --- | --- | --- | --- | --- | --- | --- | --- | --- | --- | --- | --- | --- |
|  |  | *ascV* | *hlyA* | *ela* | *ast* | *alt* | *act* | *aerA* | *pro* | *lip* | *fla* |  | FA ratio | Score |
| *A. veronii* bv. sobria | *EMA46* | *+* | *+* | *+* | *+* | *-* | *-* | *+* | *+* | *+* | *+* | **III** | *1.0 ± 0.1* | *0.11 ± 0.01* |
|  | *EMA128* | *-* | *+* | *+* | *-* | *+* | *+* | *+* | *+* | *+* | *+* | **V** | *1.1 ± 0.1* | ND |
|  | EBA104 | *-* | *-* | *-* | *+* | *+* | *-* | *+* | *-* | *+* | *+* | VI | n.d. | 0.05 ± 0.01 |
| *A. schubertii* | EBA118 | *-* | *-* | *-* | *+* | *-* | *+* | *+* | *+* | *-* | *+* | X | 0.3 ± 0.1 | ND |
|  | EMA71 | *-* | *-* | *-* | *-* | *+* | *-* | *+* | *+* | *+* | *+* | VIII | 0.1 ± 0.1 | 0.04 ± 0.01 |
|  | EBA96 | *-* | *-* | *-* | *-* | *+* | *+* | *-* | *-* | *+* | *-* | XIV | 0.2 ± 0.1 | ND |
| *A. hydrophila* | *EMA10* | *+* | *-* | *+* | *+* | *+* | *-* | *+* | *+* | *+* | *+* | **II** | *0.9 ± 0.1* | *0.10 ± 0.01* |
|  | *EBA50* | *+* | *+* | *-* | *-* | *+* | *+* | *+* | *+* | *+* | *+* | **IV** | *1.1 ± 0.1* | *0.12 ± 0.01* |
|  | EMA133 | *-* | *-* | *-* | *-* | *-* | *+* | *+* | *+* | *+* | *+* | VII | 0.3 ± 0.1 | 0.04 ± 0.02 |
| *A. caviae* | *EBA 31* | *+* | *+* | *+* | *+* | *-* | *+* | + | *+* | *+* | *+* | **I** | *1.2± 0.2* | *0.11 ± 0.01* |
|  | EMA77 | *-* | *-* | *+* | *-* | *-* | *+* | *-* | *+* | *+* | *+* | IX | 0.3 ± 0.1 | 0.06 ± 0.01 |
|  | EMA83 | *+* | *-* | *-* | *-* | *-* | *+* | *-* | *-* | *+* | *+* | XI | n.d. | 0.04 ± 0.01 |
| *A. trota* | EMA122 | *-* | *-* | *-* | *-* | *+* | *-* | *+* | *+* | *-* | *+* | XII | 0.2 ± 0.1 | 0.04 ± 0.01 |
|  | EBA7 | *-* | *-* | *+* | *-* | *-* | *+* | *-* | *+* | *+* | *+* | IX | 0.3 ± 0.1 | ND |
| *A. allosaccharophila* | EMA84 | *-* | *-* | *-* | *-* | *-* | *-* | *-* | *+* | *+* | *+* | XIII | 0.1 ± 0.1 | 0.05 ± 0.02 |
| *A. eucrenophila* | EMA131 | *-* | *-* | *-* | *-* | *-* | *-* | *-* | *+* | *-* | *-* | XV | 0.2 ± 0.1 | ND |
| *V. cholerae* (CT +ve) | 569B | - | - | - | - | - | - | - | - | - | - |  | 1.8 ± 0.2 | 0.14 ± 0.02 |
| Media (-ve control) | - | - | - | - | - | - | - | - | - | - | - |  | 0.1 ± 0.1 | 0.05 ± 0.01 |

*RIL, rabbit ileal loop assay where the fluid accumulation (FA) ratio representing the accumulated fluid (ml) per cm of ileum was determined; SMA, suckling mice assay where the score indicates the ratio of gut weight and body weight. Values indicate average ± SD (n = 3) obtained from results for each strain. Shaded rows indicate genotypes of potential enterotoxic activity. CT, cholera toxin; ND, Not Done.
